# Teaching Anatomy in the XXI Century: New Aspects and Pitfalls

**DOI:** 10.1155/2013/310348

**Published:** 2013-11-07

**Authors:** Veronica Papa, Mauro Vaccarezza

**Affiliations:** ^1^Department of Human, Social and Health Sciences, University of Cassino and Southern Lazio, Campus Folcara, Via S. Angelo, 03043 Cassino, Italy; ^2^The School of Biomedical Sciences, University of Queensland, Brisbane, QLD 4072, Australia

## Abstract

Anatomy has historically been a cornerstone in medical education regardless of nation, racial background, or medical school system. By learning gross anatomy, medical students get a first “impression” about the structure of the human body which is the basis for understanding pathologic and clinical problems. Although the importance of teaching anatomy to both undergraduate and postgraduate students remains undisputed, there is currently a relevant debate concerning methods of anatomy teaching. In the past century, dissection and lectures were its sole pedagogy worldwide. Recently, the time allocated for anatomy teaching was dramatically reduced to such an extent that some suggest that it has fallen below an adequate standard. Traditional anatomy education based on topographical structural anatomy taught in lectures and gross dissection classes has been replaced by a multiple range of study modules, including problem-based learning, plastic models or computer-assisted learning, and curricula integration. “Does the anatomical theatre still have a place in medical education?” And “what is the problem with anatomic specimens?” We endeavor to answer both of these questions and to contribute to the debate on the current situation in undergraduate and graduate anatomy education.

*Doctors without anatomy are like moles.They work in the dark and the work of their hands are mounds.*

Friedrich Tiedemann

*The foundation of the study of the art of operating must be laid in the dissecting room.*

Robert Liston

## 1. Introduction

Anatomy and dissection have long been considered a milestone of medical education; in ancient Egypt, dissection was a religious ritual required as a rite of passage to the kingdom of the dead, even if the procedure was resembling more a crude autopsy than an anatomical dissection as we intend today [1]. With the founding of the first medical school in Salerno, Italy in 1235, anatomy ascended to a prominent position in the medical curriculum and human dissection was performed as a sacramental procedure that illustrated the dissertations of revered ancient authors. During the Renaissance, with the opening of the Anatomical Theatres in Padua (1490) and Bologna (1637), anatomy was considered an artistic and spiritual exploration of life, suffering, and death. Anatomists began to dissect in order to investigate the structure of the body and produced texts illustrated with images based on their dissections [2–4]. The era of scientific human anatomy is highlighted by the publication of the main opera from Andrea Vesalius (von Wesel), the real father of modern anatomy [5].

Towards the end of 20th century, dissection was the core basis in medical education. Even today defining the exact anatomical site of a lesion is crucial for a physician to resolve a problem effectively and compassionately and therefore adequate anatomical knowledge is essential for surgeons and for anyone who performs an invasive procedure on a patient. Anatomy knowledge is also pivotal to complete a medical examination, to make a diagnosis and also to properly communicate with colleagues.

Unfortunately, anatomy as a discipline is disappearing and few new anatomists are being trained properly. Worldwide curricula reforms, which have resulted in a reduction both in the gross anatomy teaching hours and its context, lead to a serious review of the way in which anatomy is taught [6–8]. Furthermore, the abolition of anatomy demonstrator positions has deprived surgical trainees of valuable exposure to clinical anatomy; a new generation of surgeons is subsequently taking up operative responsibilities despite their poor knowledge of anatomy [8]. The majority of surgical program directors in the United Kingdom also thought that anatomy knowledge of new residents was either seriously lacking (24%) or in need of a refresher course (67%), while 52% believed that current trainees had less anatomical knowledge than those enrolled 10 years ago [9]. Furthermore, in the United Kingdom between 1995 and 2000 there was a 7-fold increase in claims associated with anatomical errors submitted to the Medical Defence Union [10]; Cahill et al. have expressed the concern that out of 80,000 avoidable deaths per year in the United States at least some can be attributed to anatomical incompetence [11]. Oliver Beahrs, an internationally acclaimed surgeon from the Mayo Clinic, and the first President of the American Association of Clinical Anatomists, quite bluntly said, “... today's residents in surgery are learning their anatomy on sick patients for the first time in the middle of the night: operating without a firm knowledge of anatomy leads to increased mortality and morbidity” [12]. Waterston and Stewart [13] gathered clinicians' opinions on this subject with a survey and their results indicate that the majority of clinicians believe that anatomy is not adequately taught, and as a result, students' knowledge is below the minimum standard required for safe medical practice.

The thorny question is why students do not seem to have enough anatomical knowledge to practice safely. The answers are various: ranging from reduced teaching hours, to recently developed teaching methods not including compulsory dissecting and light microscopy sessions. Two recent studies report that in Australian Medical Schools gross anatomy teaching had reduced from approximately 500 hours per year in its former undergraduate medical degree to an average of 52.5 hours in its new graduate medical program [7, 14]. 

More recently, medical education has experienced changes driven by evidence from the fields of psychology and education: retention of knowledge is promoted when students are actively involved in their learning and curricula integration seems to be an essential component of this process. The rationale often given for integration of the basic sciences and clinical medicine is that integration is the right way a clinician must think about when in contact with a patient.

In this context, problem-based learning (PBL) and computer-assisted learning (CAL) allow horizontal and vertical integration of different disciplines enhancing the integration of students' knowledge; moreover, using case studies, students can link clinical features with basic science concepts.

Today, as a result of this, many medical schools have incorporated active learning methods such as problem-based learning (PBL) and computer-assisted learning (CAL) into their courses [15] where the main feature is the integration of different basic science disciplines in one course. Although a shift is clearly visible from ‘‘traditional” teacher-centerd education, with students as passive recipients of information, to ‘‘innovative” student-centerd education, concern is rising about the level of knowledge achieved by students graduating from innovative programs, for basic sciences in general, and for anatomy in particular [16].

In recent studies on curriculum integration and different strategies to implement it [17, 18], it has been reported that, although curriculum integration at the beginning of medical training made perfect sense, this skill is not intuitive to many students, mainly first year students, who fail to see how various concepts from different sciences could fit together. The proposal was to integrate basic science in the first year and clinical science during the second so that the knowledge acquired in the first year classes could be applied in problem solving and critical thinking in the following years of training.

However, studies on the outcomes of PBL and CAL have shown contradictory results [19–22]. In a more recent study by Van der Vleuten group [23], students divided into tutorial groups according to PBL program policy tended to skip the causes and the underlying mechanisms of a case study they were asked to deal with and to immediately start looking for the correct diagnosis and not to bother to formulate appropriate learning objectives. More importantly O'Neill [24], unlike others [25], suggests that the focus on diagnostic problems should inhibit the building of an appropriate knowledge of basic sciences compulsory for medical graduate to safely practice.

Van der Vleuten group, in a study comparing Dutch students' levels of anatomy knowledge as measured by a case-based anatomy test with standards set by different groups of experts, also reported that many students did not know enough about anatomy as the standards established by the anatomists, clinicians, and recent graduates would yield failure rates of 42%, 58%, and 26%, respectively [26].

In fact, the integrated PBL approach seems to be associated with uncertainty among students about their basic science knowledge as well as presumed deficiencies particularly in clinical anatomy [23].

On the other hand, Gogalniceanu et al. [27] studied 174 first and second year London medical students and observed that 99% of the students both agreed that more curricula time was needed to understand the subject they were expected to learn and disapproved of the proposal to close the university's dissection facilities and remove dissection from the curriculum.

Dissection and prosection were considered to be the most useful methods of learning anatomy (75% of students believed dissection was the single most useful method of learning anatomy), whilst the least popular was the PBL/CAL combined.

*Is Using Human Body Donation in Medical Education Therefore Old-Fashioned or Not?* As previously stated [2–4], dissection is intimately bound to the study of the human body and the medical training; in the Anglo-Saxon countries, according to Anatomy Act of 1832 (which was subsequently repealed by the Anatomy Act in the 1984 and by the Human Tissue Act in the 2004), the traditional view is, so far, that teaching using anatomic specimens will provide the essential building blocks of knowledge for future doctors.

However, just as the use of human tissue for research has become controversial [28] for ethical and practical reasons, the use of human specimens for teaching purposes is surrounded by emotional and ethical worries [29, 30]. Nevertheless, Lempp [31] studied the reactions and outcomes that undergraduate medical students described about their own experiences in dissection and found that the majority of the students appeared to have enjoyed and really appreciated the close personal supervision of the anatomy demonstrators and the more experienced permanent anatomy staff as well as the opportunities to learn together in small groups.

These results, together with other studies [14, 27, 32], reveal that the old-fashioned view that dissection is an emotionally challenging way to learn anatomy needs to be reconsidered.

Dissection and light microscopy are, though, not free of handicaps. Storing human bodies is expensive, and other issues such as preservation and reduced suitability for dissection due to illness, elderly, or obesity could be a problem; careful dissection is time-consuming, and light microscopes are expensive to maintain, especially nowadays where all that matters is funding basic molecular research while financing knowledge and training seems to be anachronistic and useless. 

However, through performing dissection/prosection and light microscopy, students could learn the best about the surrounding tissues and structures while seeking a particular nerve or muscle; hands-on teaching with anatomic specimens is the first experience of the structural organization of the body and leads to a real understanding of the three-dimensional configuration of patients' anatomy.

Dissection has its obvious limitations, such as not being useful for teaching various important areas such as skeletal, nervous system anatomy or muscular anatomy in the contracted state. For these lectures, alternative tools would be required, such as cleaned and articulated skeletal models, radiological films, plastinated models, computer simulations, and Thiel-method embalmed bodies (see the following).

However, it is essential to provide students with the best evidence of biological variation: two individuals are not necessarily the same anatomically; as students wander from one body to the next in the dissecting room, they will see anatomical variation associated with developmental anomalies which often are common and of clinical importance.

Nowadays, the availability of human specimens for dissection is likely to be derived from a homogeneous population, mainly donated bodies of the elderly, suffering from degenerative diseases, possibly causing the onset of bias and mistakes not representative of “normality” but rather representative of “reality” of the ongoing ageing of the whole population.

Of note, plastination is a relatively new advancement in anatomy science, an effective technique of tissue preservation of entire organs or cross-sectional body slices [33], introduced by Von Hagens et al. [34, 35]; this technique uses polymers such as resin and silicone in order to create life-like specimens. Plastination, therefore, allows realistic visualization of anatomical concepts that are simply too difficult to describe while maintaining the bodies' natural biological variance or pathology that, instead, plastic models lack. 

The University of Warwick was the first in the UK to teach with plastinated prosections (Warwick Medical School, 2009) followed by other medical schools including St George's (London, UK) and Nottingham University (UK) [36]. Student satisfaction and acceptance has also been recorded: a recent study by Fruhstorfer et al. [35] examines students' views on the use of plastinated prosections for their anatomical learning. The majority of students (94%) rated plastinated prosections as a useful tool in anatomical training and appreciated the detailed view of relevant anatomy and the relations between structures. However, learning with plastinated prosections was perceived to be compromised because of limitations in terms of tactile and emotional experience.

Conversely, some studies reveal that students who were trained only on prosection or plastination believe their anatomical knowledge to be misleading and ineffective [37].

Lastly, a new method of embalming bodies named Thiel method is worth mentioning [38]. This technique, developed by the late Professor Walter Thiel at the University of Graz (Austria), is still not widely known and it needs careful procedures and dedicated structures [39–41], but it looks promising to study in a more physiological and dynamic way the skeletal muscle system and it is reported to be one of the best training tools in the field of anatomical surgery [40, 41].

## 2. Conclusions

In an analysis of teaching and learning, it is necessary to examine the curriculum, the mode of teaching, the quality of how it is delivered, and the infrastructure within which it is delivered. 

We therefore strongly suggest anatomy to be integrated vertically into medical education so that students are exposed to anatomy teaching throughout undergraduate (preclinical and clinical), postgraduate, and later in professional training. Modern digitalized methods of teaching anatomy are undoubtedly useful. However, body donation can still significantly benefit new medical students, and the dissection procedures should be reintegrated into medical training. 

Computer assisted learning, problem based learning, and newly developed techniques such as plastination should be used to enhance and support anatomical teaching and learning in medical education. 

We are confident that gross anatomy through dissection and prosection cannot be undermined in a modern medical curriculum, since it gives a 3D experience in real life that cannot be attained by the most advanced digital anatomy programs available.

The new digital tools and the new PBL and integrative teaching methods have to be ancillary and complement the gross anatomy education and the lecture experience; the modern teaching of anatomy encompasses these paths as well as the most advanced teaching and learning techniques that comprehend the most efficacious pedagogic methods proven to maximize the teacher activity and the learner performance [42–44]. 
